# Intentional Pencil Ingestion – A Case Report of a Sharp Ingested Foreign Body

**DOI:** 10.5070/M5.53005

**Published:** 2026-10-31

**Authors:** Francis Gan, Nathan Mercado, Abhijnya Gowder, Daniel Ng, Sassan Ghassemzadeh, John Costumbrado

**Affiliations:** * Riverside Community Hospital, Department of Emergency Medicine, Riverside, CA; ^ University of California, Riverside, School of Medicine, Riverside, CA

## Abstract

Treating foreign body ingestion in the emergency department (ED) is common; however, certain ingestions may be immediately life-threatening, depending on the object because sharp objects pose the greatest risk for gastrointestinal injury, including perforation and obstruction.^1^ This is a case of an adult male who arrived at the ED with suicidal ideation and reported ingesting a pencil two days prior. He presented with abdominal pain and hematochezia. A computed tomography (CT) scan of the abdomen and pelvis identified the pencil in the distal ileum. The patient was admitted for surgical evaluation and monitoring. Remarkably, the pencil was passed spontaneously in the stool on hospital day six without signs of colonic perforation. This case illustrates how selected cases of high-risk foreign body ingestion may be managed conservatively, though close monitoring is critical given the risks of perforation, obstruction, or failure of progression.

**Topics:**

Abdominal pain, CT, foreign body ingestion, mental health.

**Figure:**
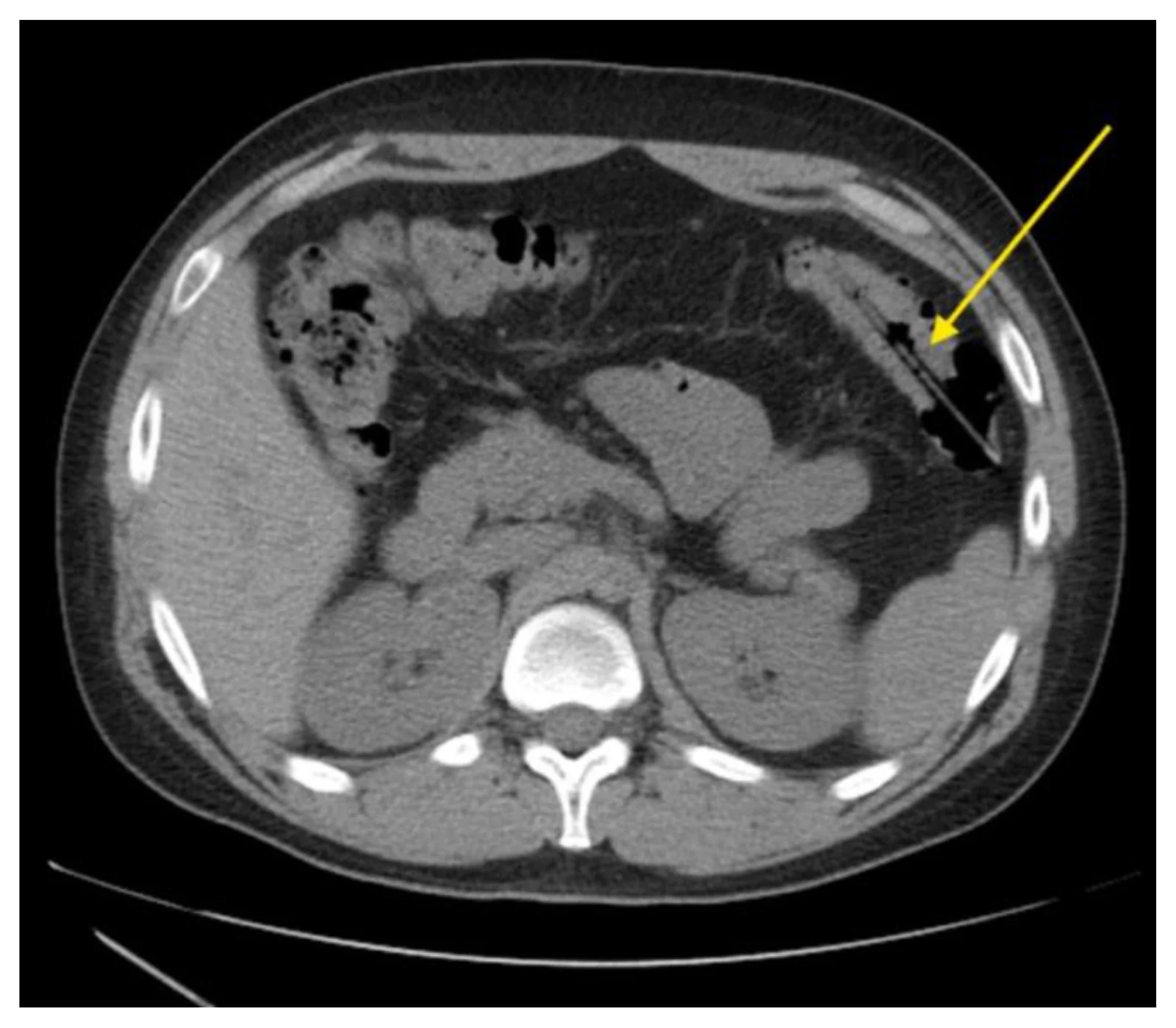


**Figure:**
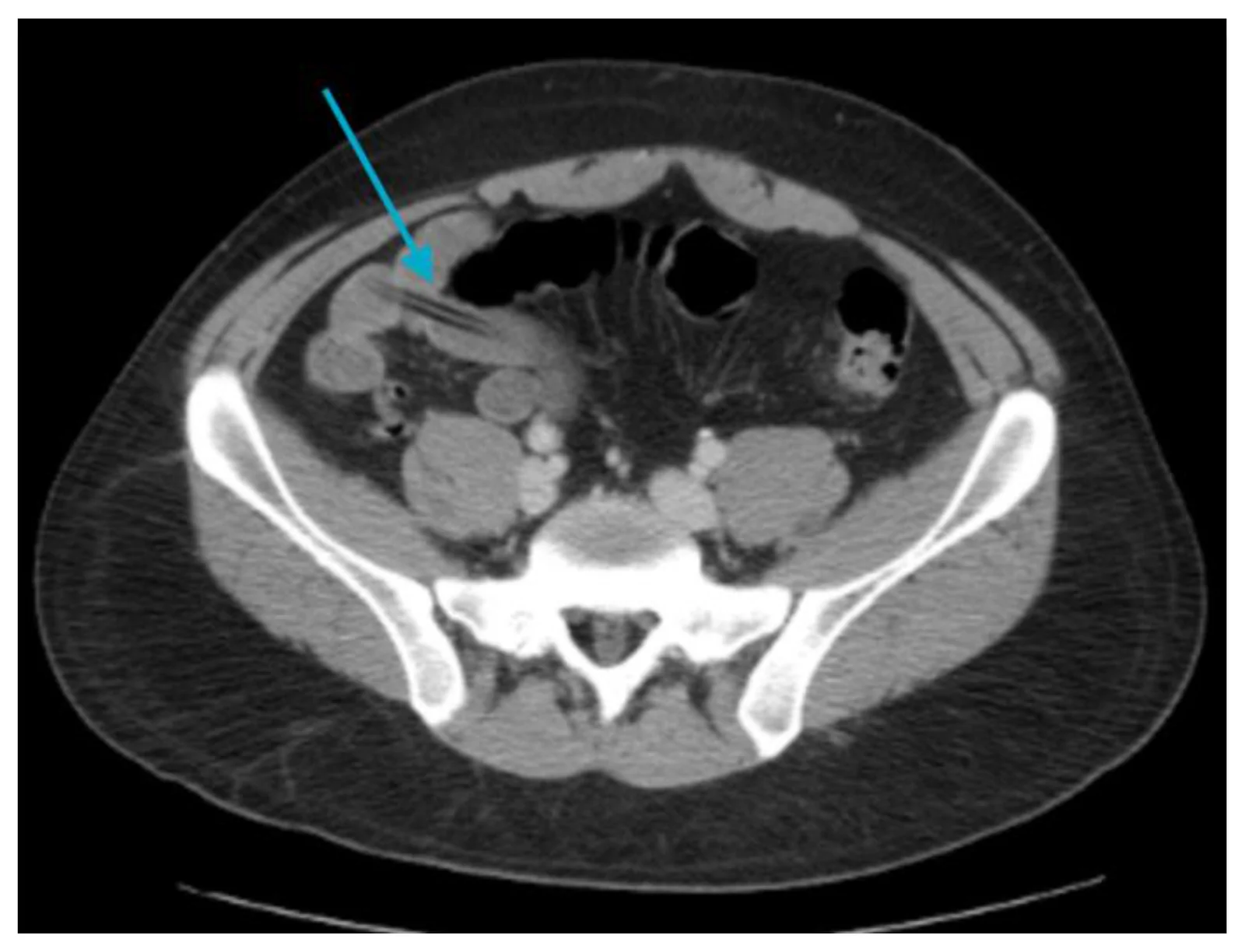


**Figure:**
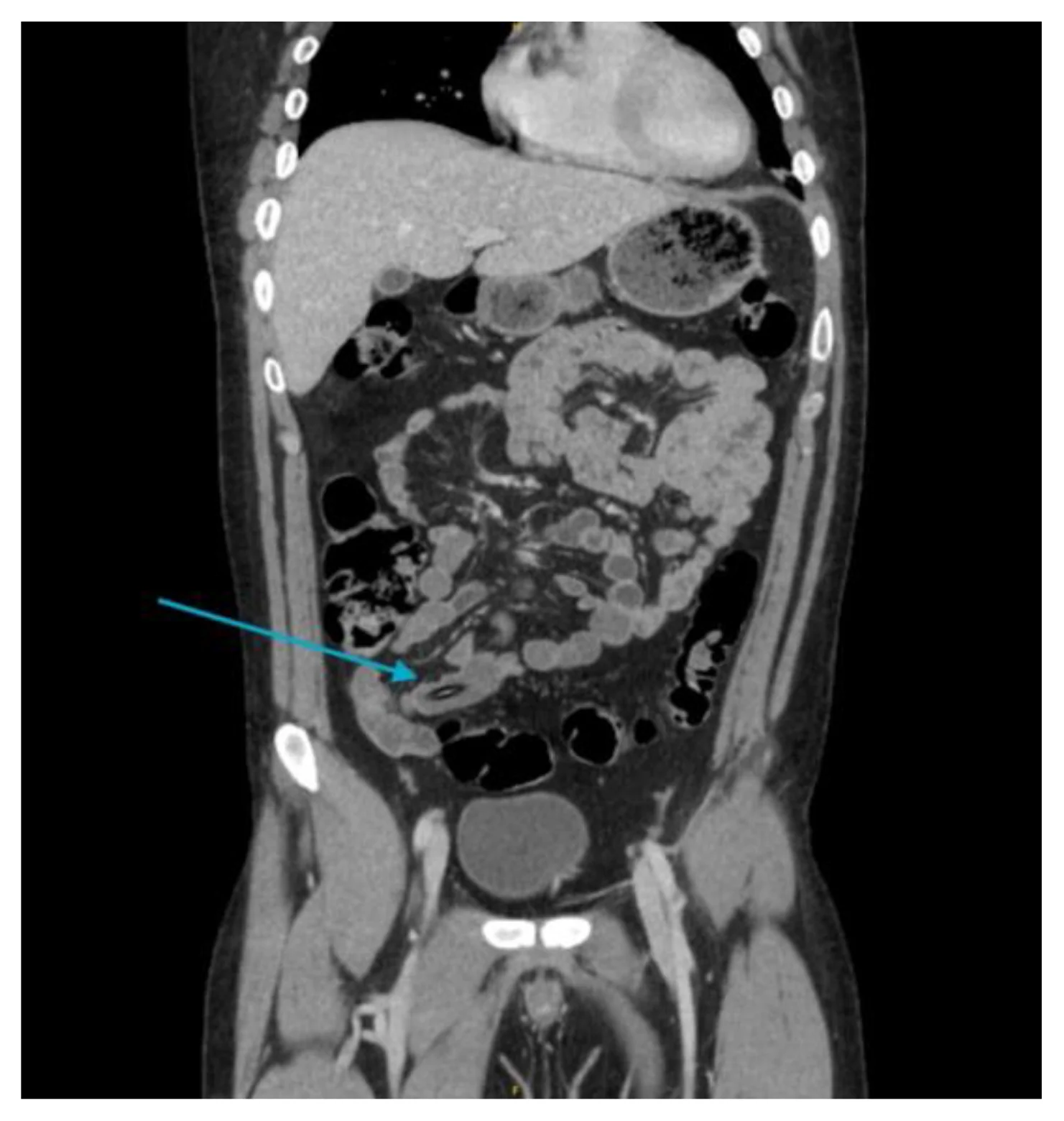


## Brief introduction

Foreign body ingestion is commonly observed in the pediatric population, typically occurring accidentally over intentionally. Most cases resolve spontaneously without intervention.^1^ Deliberate foreign body ingestion (DFBI), a rare form of self-harm, is more commonly seen in patients with psychiatric disorders.^2^ Depending on the nature of the ingested object, particularly those with sharp edges or made of glass or metal, there is an increased risk of gastrointestinal (GI) injury, including obstruction, mucosal irritation, laceration, and perforation. Ingestion of long foreign bodies, such as pencils, is exceedingly rare, with only a few documented cases in the literature.^3^ Management of such cases can vary widely, from conservative observation to surgical intervention, including exploratory laparotomy. Despite its rarity, clinicians should maintain a high index of suspicion for GI complications in cases of foreign body ingestion. This case report details the evaluation and management of a 23-year-old male patient who presented following intentional ingestion of a pencil.

## Presenting concerns and clinical findings

A 23-year-old male presented to the emergency department (ED) with concerns of foreign body ingestion after swallowing a pencil two days prior. The patient reported experiencing visual and auditory hallucinations which led him to ingest the pencil as an act of self-harm. Upon evaluation, he endorsed generalized abdominal pain and reported passage of bright red blood per rectum.

## Significant findings

Written consent for publication was obtained from the patient. Computerized tomography (CT) of the abdomen and pelvis two days after the ingestion of the pencil revealed a hypodense foreign object measuring 7.9 cm (blue arrows) in the lumen of the distal ileum. The images obtained demonstrate that the object progressed past physiological strictures within the gastrointestinal tract such as the esophagus and stomach; however, it had yet to progress through the ileocecal junction. Ingested foreign bodies carry a high risk of obstruction and perforation at these physiological stricture points. A repeat CT scan six days later showed that the pencil (yellow arrow) had progressed to the distal transverse colon.

## Patient course

The patient initially presented to the ED for abdominal pain and rectal bleeding. Given the patient’s presentation and imaging, there was concern for an increased risk for perforation and/or obstruction. General surgery and gastroenterology were consulted for possible intervention. The patient was admitted with a plan to observe for continued progression of the foreign body. On repeat imaging six days later, the pencil had progressed to the distal transverse colon and the patient passed the pencil in their stool later that afternoon. The patient was deemed medically stable but given the concern for self-harm, arrangements were planned for the patient to be transferred to a psychiatric facility for further evaluation and management.

## Discussion

Ingestion of foreign objects in adults is relatively uncommon but can occur in specific populations such as the elderly, individuals with alcohol use disorder, or those with psychiatric conditions. The management of foreign bodies can also be costly, since the average cost of the associated hospital admission has been estimated to be approximately $14,000.^4^ Although approximately 80% of ingested materials pass spontaneously, further evaluation is often required to determine appropriate management, which may range from observation to surgical intervention.^5^ Key considerations include the size and shape of the object, time since ingestion, its location at the time of evaluation, and any associated symptoms. Sharp or irregularly shaped objects, those containing magnets or harmful chemicals such as batteries, are strong indications for endoscopic removal.^1^ Imaging plays a critical role in localizing foreign objects, assessing their characteristics, and guiding management strategies, particularly when patients are unable to provide reliable histories.^6^ For example, CT is often used for the diagnosis of esophageal foreign bodies with a higher sensitivity (97%) and specificity (98%) compared to plain films (47% and 52%, respectively).^7^ Foreign bodies are most likely to become lodged at four anatomical sites within the digestive tract: the lower esophageal sphincter, the pylorus of the stomach, the C-loop of the duodenum, and the ileocecal junction.^3^ Management strategies vary based on the location of the object. If the object is located in the stomach, it is likely to pass without intervention. However, removal is indicated if it remains in the pylorus for three to four weeks, in the duodenum for four to eight days, or beyond the duodenum for more than four days.^8^ In a retrospective analysis of ingested foreign bodies, it was found that 30.8% of surgical interventions were performed for long objects (>6 cm) and 15.4% for sharp objects causing perforation. Obstruction at the ileocecal region accounted for 53.8% of cases requiring surgery, irrespective of the size or shape of the ingested object.^9^

In this case, a patient with a psychiatric history arrived at the emergency department reporting the ingestion of a 7.9 cm sharpened pencil two days prior, and he had rectal bleeding and abdominal pain. Imaging revealed the object in the distal ileum upon arrival, and it was passed six days later without evidence of perforation. According to standard management guidelines, surgical removal would have been warranted given that the object remained beyond the duodenum for more than four days. However, additional factors must be considered in psychiatric populations.

Psychiatric illness can add to complexity in treatment with the challenges associated with assessing capacity to make decisions and obtaining informed consent.^4^ When objects have the potential to pass spontaneously, especially once beyond the duodenum, conservative management may be preferred to avoid reinforcing maladaptive behaviors or secondary gain, such as attention-seeking. This approach, however, necessitates continuous communication between the surgical, medical, and psychiatric teams to ensure appropriate care and prevention of future occurrences.^10^

## Supplementary Information












